# Clinical and microbiological factors predicting outcomes of nonfermenting gram-negative bacilli peritonitis in peritoneal dialysis

**DOI:** 10.1038/s41598-021-91410-0

**Published:** 2021-06-10

**Authors:** Ana Claudia Moro Lima dos Santos, Rodrigo Tavanelli Hernandes, Augusto Cezar Montelli, Aydir Cecília Marinho Monteiro, Thais Alves Barbosa, Carlos Henrique Camargo, Adriano Martison Ferreira, Alessandro Lia Mondelli, Maria de Lourdes Ribeiro de Souza da Cunha, Pasqual Barretti

**Affiliations:** 1 Biosciences Institute of Botucatu, Sao Paulo State University Julio de Mesquita Filho (UNESP), Botucatu, Brazil; 2 Adolfo Lutz Institute, São Paulo, Brazil; 3 Botucatu Medical School Clinics Hospital, Botucatu, Brazil; 4 Botucatu Medical School, Sao Paulo State University Julio de Mesquita Filho (UNESP), Botucatu, Brazil

**Keywords:** Microbiology, Nephrology

## Abstract

Peritonitis due to gram-negative bacilli (GNB), particularly nonfermenting GNB (NF-GNB), is a serious complication of peritoneal dialysis with a low resolution rate. Beyond the patient’s condition, microbiological properties such as antimicrobial resistance, biofilm production and other virulence factors can explain the poor outcomes. This study aimed to evaluate the influence of patient condition, microbiological characteristics, including biofilm production, and treatment on peritonitis outcome. We reviewed the records of 62 index episodes caused by NF-GNB that occurred between 1997 and 2015 in our center. The etiologies were species of *Pseudomonas* (51.6%), *Acinetobacter* (32.2%), and other NF-GNB (16.1%). There was a high (72.9%) proportion of biofilm producer lineages. The in vitro susceptibility rate of *Pseudomonas* spp. to amikacin, ciprofloxacin, and ceftazidime was significantly greater than that of *Acinetobacter* spp. and other species; however, there was a similar low resolution rate (< 45%) among the episodes attributable to them. Preexisting exit-site infection was independently associated with nonresolution. No other factor, including biofilm production, was associated with the outcome. The higher in vitro susceptibility of *Pseudomonas* compared to other NF-GNB that presented a similar resolution rate suggests that bacterial virulence factors such as biofilms can act in concert, thereby worsening the outcome.

## Introduction

Continuous peritoneal dialysis (PD) was introduced in the 1970s^1,2^, and its initial results were compromised by the high incidence of bacterial peritonitis^3,4^. Since then, technological advances, particularly in disconnection systems and antimicrobial prophylaxis, have strongly reduced the incidence of these infections^5,6^. However, peritonitis remains a serious complication of PD and the main cause of PD failure and is associated with a higher risk of death from all causes and cardiovascular causes^7,8^.

Gram-positive cocci are the main etiology of PD peritonitis worldwide, while episodes due to gram-negative bacilli (GNB) usually present greater severity and lower resolution rates^9,10^. Among them, the worst outcomes are reported in infections caused by *Pseudomonas* species and other nonfermenting GNB (NF-GNB)^11–13^. The findings of a large prospective Brazilian cohort showed that *Pseudomonas* spp. etiology is independently associated with the nonresolution of peritonitis^14^.

The reasons for the unfavorable evolution of NF-GNB peritonitis are not fully known. Beyond the patient’s clinical and demographic characteristics and antibiotic treatment, factors associated with intrinsic bacterial virulence and antimicrobial resistance are possible determinants of worse outcomes^13,15–19^.

NF-GNB are ubiquitous and opportunistic microorganisms that are present in nature and the healthcare environment, where they cause different types of infections^20,21^. *Pseudomonas* spp. are the most isolated NF-GNB and of greatest clinical importance. *Pseudomonas* species virulence factors enable them to invade tissues, proliferate rapidly, generate biofilms, quickly develop antibiotic resistance and provide those species with great motility^16–19^. *Acinetobacter* species have been an increasing concern in PD due to their alarming rate of antibiotic resistance development; in particular, the *Acinetobacter baumannii* complex^13^ can form biofilms and colonize catheters^22^.

In turn, only a few studies have reported the factors influencing the outcomes of NF-GNB-induced PD-related peritonitis, highlighting a study by Silva et al.^11^, who reported that the use of two antimicrobial agents favored positive outcomes in *Pseudomonas*-induced peritonitis.

Jointly analyzing the microbiological properties of the causative organism, patient-related conditions, PD modality, and peritonitis episode characteristics and its treatment can potentially identify the determinants of outcomes in NF-GNB peritonitis, but such an analysis has not been conducted in Brazilian or Latin American cohorts. Therefore, the present study aimed to investigate whether causative bacterial characteristics, including the ability to produce biofilms, as well as those of the patient, PD modality, peritonitis episode, and peritonitis treatment, influenced the clinical evolution of NF-GNB-induced PD-related peritonitis.

## Results

### Study population

**Figure 1:**
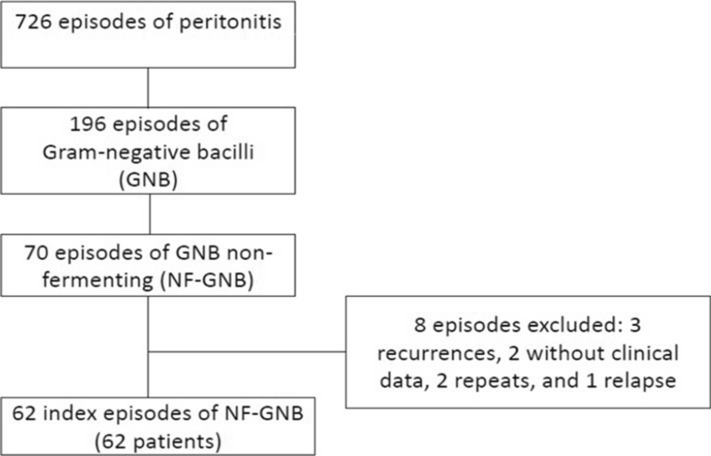
Flow diagram showing from the total peritoneal-related bacterial peritonitis episodes to index episodes caused by nonfermenting gram-negative bacilli.

Between June 1997 and December 2015, there were 726 episodes of bacterial peritonitis in 542 PD patients in our center. Of these, 194 (26.7%) were caused by GNB, 70 of which were caused by NF-GNB. Based on the exclusion criteria, we studied 62 index cases of peritonitis caused by NF-GNB from 62 adult patients (Fig. 1). No episodes were caused by polymicrobial organisms. Preexisting ESI was diagnosed in 16 cases (25.8%): nine were caused by *Pseudomonas aeruginosa*, three by *Burkholderia cepacia,* one by *Stenotrophomona maltophilia*, one by *Acinetobacter baumanii*, and two by *Corynebacterium* spp. These same organisms were seen in peritoneal effluent culture in 12 cases. For all ESI episodes, we prescribed oral ciprofloxacin as the initial treatment. No tunnel infection was diagnosed by clinical criteria, although we did not perform ultrasonographic evaluation of the subcutaneous catheter tunnel. Previous peritonitis caused by other bacteria was reported in 23 cases (37.1%). The clinical and demographic data of the 62 patients at the time of their first episode of peritonitis are shown in Table 1.

**Table 1:** Characteristics of 62 patients the time of the 1st episode of nonfermenting Gram-negative bacilli peritonitis.

Characteristics	Mean ± standard deviation/number (%)
**Age (years)**	45.4 ± 20.4
≤ 60	44 (70.9)
> 61	18 (29.1)
Gender (male)	35 (55.6)
Caucasians	46 (73.0)
**Underlying kidney disease**	
Diabetes renal disease	16 (25.8)
Hypertensive nephrosclerosis	20 (32.2)
Glomerulonephritis	7 (11.3)
Autosomal recessive polycystic kidney disease	3 (4.8)
Undetermined and others	16 (25.8)
**Topical antibiotic use at the catheter exit-site**	
None	27 (43.5)
Mupirocin cream	16 (25.8)
Gentamycin cream	19 (30.6)
PD vintage (months)	15.4 ± 20.5
**Dialysis mode**	
Continuous ambulatory peritoneal dialysis (CAPD)	32 (51.6)
Automated peritoneal dialysis (APD)	30 (48.4)

### Treatment of a peritonitis episode

All patients started treatment within 24 h of the onset of clinical signs or symptoms of peritonitis, based on ISPD guidelines^23^. From 1996 to 2000 (16 cases), the initial antibiotic therapy consisted of intraperitoneal (i.p.) cefazolin (20 mg/kg daily) plus amikacin (2 mg/kg daily). From 2000 to 2005, we used two regimens: the first (four cases) consisted of i.p. cefazolin (20 mg/kg daily) plus amikacin (2 mg/kg daily), and the second (nine cases) was i.p. cefazolin (20 mg/kg daily) plus ceftazidime (1–1500 mg daily). After 2005 (33 cases), the initial treatment for all was i.p. vancomycin (15–30 mg/kg every 5–7 days) plus amikacin (2 mg/kg daily). The dosages were adapted for APD patients, according to the ISPD guidelines^23^. If there was no improvement after 48 h, cell counts and repeat cultures were performed^23^. When the results of peritoneal effluent culture and in vitro susceptibility tests were available, we adjusted the treatment. For the episodes caused by *Pseudomonas* spp*.* and *Stenotrophomonas,* we used two effective (bacterial susceptible) antipseudomonal antibiotics, according to the ISPD guidelines^23^; for the others, the adjustment was made based on “in vitro susceptibility”. Therefore, for episodes caused by *Pseudomonas* spp*.,* we adjusted them as follows: two antipseudomonal drugs in 28 cases (amikacin plus ciprofloxacin in 20 and amikacin plus ceftazidme in eight episodes), monotherapy in three cases, with imipenem, ceftazidime, and ciprofloxacin, and no adjustment in one episode caused by a multiresistant strain in which immediate catheter removal occurred. We had an episode of *Stenotrophomonas maltophilia* in which we used ceftazidime plus ciprofloxacin for adjustment. The duration of antibiotic therapy was 21 days for *Pseudomonas* spp. and 28 days for *Stenotrophomonas maltophilia* episodes^23^. For the 20 *Acinetocbacter* spp*.* episodes, we adjusted based on monotherapy with imipenem in nine, amikacin in 10, and ceftazidime in one. Considering the good stability of the antibiotics above at room temperature or in a refrigerator^23^, therapy was provided at the patient’s home by the patients themselves or their caregivers.

The duration of antibiotic therapy was 21 days.

### Etiologies and in vitro susceptibility

According to routine microbiological tests, we observed the following in vitro susceptibility rates: (1) *Pseudomonas* spp*.* (n = 32)*:* amikacin = 75%, ciprofloxacin = 71.9%, ceftazidime = 81.2%, and imipenem = 90.6%; (2) *Acinetobacter* spp*.* (n = 20): amikacin = 50%, ciprofloxacin = 40%, ceftazidime = 45%, and imipenem = 90%; and (3) others: (n = 10): amikacin = 30%, ciprofloxacin = 20%, ceftazidime = 50%%, and imipenem = 70%.

The descriptions of the etiological agents are shown in Tables 2 and 3. Of the total episodes of peritonitis included in the study, special microbiological tests were carried out in 48 episodes (Table 3), as it was not possible to recover the other strains.

**Table 2:** Etiologic spectrum of 62 non-fermenting Gram-negative bacilli peritonitis episode.

	Episodes n (%)	Recovered isolates (n)
***Pseudomonas* spp.**	32 (51.6)	24
*Pseudomonas aeruginosa*	28 (45.2)	22
*Pseudomonas putida*	2 (3.2)	2
*Pseudomonas fluorescens*	2 (3.2)	0
***Acinetobacter* spp.**	20 (32.2)	18
*Acinetobacter baumanii*	12 (19.3)	11
*Acinetobacter haemolyticus*	5 (8.1)	5
*Acinetobacter lwoffi*	1 (1.6)	0
*Acinetobacter ursingii*	2 (3.2)	2
***Burkholderia* spp.**	5 (8.1)	2
*Burkholderia cepacia*	4 (6.4)	1
*Burkholderia gladioli*	1 (1.6)	1
***Achromobacter* spp.**	4 (6.4)	3
*Achromobacter denitrificans*	3 (4.8)	3
*Achromobacter xylosoxidans*	1 (1.6)	0
***Stenotrophomona* spp.**	1 (1.6)	1
*Stenotrophomona maltophilia*	1 (1.6)	1
Total	62 (100)	48

**Table 3:** Non-fermenting Gram-negative bacilli-causing peritoneal dialysis-related peritonitis episodes and their in vitro susceptibility rates by minimal inhibitory concentration.

	*Pseudomonas* spp. (n = 24)	*Acinetobacter* spp. (n = 18)	*Achromobacter* spp.(n = 3)	*Burkholderia gladioli* (n = 1)	*Burkholderia cepacia* (n = 1)	*Stenotrophomonas* spp. (n = 1)	NF-GNB (n = 48)
	Susceptibility n (%)	Susceptibility n (%)	Susceptibility n (%)	Susceptibility n (%)	Susceptibility n (%)	Susceptibility n (%)	Susceptibility n (%)
Amikacin	20 (83.3)^a,b^	7 (38.9)	1 (33.3)	1 (100)	–	–	29 (60.4)
Ciprofloxacin	17 (70.1)^a^	7 (38.9)	2 (66.7)	0 (0.0)	–	–	26 (54.1)
Ceftazidime	21 (87.3)^a^	8 (44.4)	3 (100)	0 (0.0)	1 (100.0)	1 (100.0)	39 (70.8)
Cefepime	20 (83.3)^a,b^	7 (38.9)	1 (33.3)	0 (0.0)	–	–	28 (58.3)
Imipenem	20 (83.3)	16 (88.9)	3 (100)	0 (0.0)	–	–	39 (81.2)

Data refers only to 48 recovered isolates. Statistical comparisons (Chi Square test).

^a^*p* < 0.05 versus *Acinetobacter* spp.

^b^*p* < 0.05 versus *Achromobacter* spp.

### Special microbiological tests

The results of in vitro susceptibility based on the minimal inhibitory concentration by E-test are described in Table 3. *Pseudomonas* species were more susceptible than *Acinetobacter* species to all the tested antimicrobials, except imipenem. *Pseudomonas* species were also more susceptible than *Achromobacter* species to amikacin, ciprofloxacin, and cefepime. Isolates of *Burkholderia cepacia* and *Stenotrophomonas maltophilia* were tested only for ceftazidime, and all were susceptible.

Regarding biofilm production, of the 48 samples, 35 (72.9%) produced biofilms. There were 18 strong producers, seven medium producers, and 10 weak producers. The biofilm producers were 22 of the 24 *Pseudomonas* isolates, 11 of the 18 *Acinetobacter* isolates, and one of the three *Achromobacter* isolates. Both *Burkholderia cepacia* and *Burkholderia gladioli* isolates were not producers, while the only isolate of *Stenotrophomonas maltophilia* was a producer. Among the episodes caused by biofilm, there were nine cases with ESI and seven with ESI caused by the same organism found in the dialysate culture, while among those by nonproducers, there were four. There was only one resolution in the first and second groups of episodes and no resolution in the third group (*p* > 0.99).

### Outcomes

Twenty (32.2%) of 63 NF-GNB peritonitis episodes resolved. Regarding the etiology and the outcomes, there was resolution in 10 (31.2%) of the infections caused by *Pseudomonas* species, 8 (40%) of the cases caused by *Acinetobacter* species, and two (22.2%) caused by other NF-GNB (*p* = 0.39).

### Factors associated with peritonitis outcome

#### Univariate analysis

Univariate logistic regression analysis revealed that preexisting ESI, age, resistance to ceftazidime, and initial treatment with cefazolin plus ceftazidime were associated with a higher risk of nonresolution of peritonitis at a *p* value < 0.20 (Table 4). The other variables (underlying kidney disease, sex, race, PD modality, dialysis vintage, topical antibiotic use, previous peritonitis due to other bacteria, biofilm production, resistance to amikacin, initial treatment, and subsequent antibiotic regimen adjustment) did not reach *p* < 0.20 and were not included in the multivariate model. There was collinearity between resistance to ceftazidime and initial treatment; therefore, it was not possible to include them together in the same regression model.

**Table 4:** Predictors of non-resolution of non-fermenting Gram-negative bacilli peritonitis in peritoneal dialysis—Logistic Regression Analysis.

Variable	*p* value (univariate)	*p* value (multivariate)	Odds ratio	95% CI
Age (years)	0.135	0.088	1.026	0.977–1.057
Exit-site infection (yes)	0.027	0.021	12.75	1.469–89.757
Resistance to ceftazidime	0.171	0.98	5.50	0.634–47.74

#### Multivariate analysis

This analysis showed that only preexisting ESI was an independent predictor of nonresolution (Table 4). We constructed a second model (model 2), in which we included the covariate initial treatment cefazolin plus ceftazidime and removed resistance to cefatzidime, and observed a tendency for the protocol of cefazolin plus ceftazidime (using other initial treatments as a reference) to be associated with nonresolution, retaining preexisting ESI as a predictor of nonresolution (Table 5).

**Table 5:** Predictors of non-resolution of non-fermenting Gram-negative bacilli peritonitis in peritoneal dialysis—Logistic Regression Analysis (model 2).

Variable	*p* value (univariate)	*p* value (multivariate)	Odds ratio	95% CI
Age (years)	0.135	0.082	1.030	0.996–1.065
Preexistent exit-site infection (yes)	0.027	0.017	18.87	1.703–110.76
Initial treatment with cefazolin plus ceftazidime (vs other treatments)	0.122	0.097	5.33	0.512–10.63

Finally, we performed a post hoc subanalysis where in model 2, we replaced the variable preexisting ESI with preexisting ESI caused by the same organism (n = 12) found in the dialysate culture, and the latter was also an independent predictor of nonresolution (OR = 10.8, 95% CI 1.3–89.3, *p* = 0.027).

## Discussion

In human infections, the clinical course and outcome are strongly dependent on the characteristics of the infecting microorganism and the patient’s condition. In the case of bacteria, despite the indisputable role of bacterial resistance, this does not seem to be the only property influencing the outcomes; this holds true for PD-related peritonitis. Previous publications by our group showed a high number of virulence factors among *Staphylococcus aureus* lineages, some of which are associated with worse PD-related peritonitis outcome, despite their low resistance rate to methicillin^24,25^. In a previous report on PD-related *Escherichia coli* peritonitis episodes, we did not find any resistant strain to amikacin or ceftazidime used as initial treatment; in contrast, only 48.1% of the episodes progressed to resolution^26^. In the same study, approximately 50% of the isolates were medium or strong biofilm producers, which tended to be associated with nonresolution, but this did not reach statistical significance (*p* = 0.09)^26^.

NF-GNB presents both a high antimicrobial resistance rate and high production of virulence factors, such as biofilms, as confirmed in the present series, which potentially could explain the low observed resolution rate. In addition, important virulence factors are present in NF-GNB, particularly *Pseudomonas* species, which induce bacterial adhesion, destruction of cell membranes, and inhibition of the macrophage response, in addition to other actions^16–19,27,28^^.^

Interestingly, we did not find a high resistance rate among episodes caused by *Pseudomonas* species, approximately 80% of which were susceptible to frequently used antimicrobials such as amikacin and ceftazidime. Even so, the resolution rate of these episodes was just over 30%, similar to that observed with peritonitis caused by other NF-GNB, which suggests the influence of other factors on the outcome. Moreover, the adjustment of the initial antibiotic regimens, based on etiologies and their in vitro susceptibility, did not have an association with the outcome. On the other hand, over 90% of the *Pseudomonas* isolates were biofilm producers. We emphasize that antibiotic susceptibility is based on the MIC of the drug for planktonic cells, which are more sensitive to antimicrobials than cells wrapped in biofilms^29^.

The aggressive character of *Pseudomonas* spp. can explain, at least partially, the findings of this and of the two largest studies, which previously described peritonitis caused by these bacteria as the most frequent etiology of peritonitis among NF-GNB. Siva et al.^11^ studied 191 episodes of *col* peritonitis that occurred in Australian patients who reported high rates of catheter removal (44%), permanent hemodialysis transfer (35%), hospitalization (96%), and a change to a second antibiotic (66%). Lu et al.^12^ reviewed 153 episodes of peritonitis caused by *Pseudomonas* species in Hong Kong, reporting an overall primary response rate of 53.6% and a complete cure rate of 42.4%. Interestingly, that study showed a decrease in the incidence of germs resistant to ceftazidime and gentamicin over time.

A significant number of NF-GNB-induced peritonitis cases involved *Acinetobacter* species. Of these, the majority were due to *Acinetobacter baumannii*, similar to previous reports^13,30^. The importance of these bacteria has increased in recent years due to their great capacity to acquire mechanisms of resistance to different classes of antibiotics, great ability to survive and adapt to adverse conditions, and ability to adhere to different surfaces by the formation of biofilms^31^. This series confirms that *Acinetobacter baumannii* is resistant to several antimicrobials, except imipenem. As expected, over 50% of these strains were biofilm producers. Despite their greater bacterial resistance compared to *Pseudomonas* spp*.*, the resolution rate was similar between them. In this way, we can speculate that virulence factors influenced the outcome.

Other identified germs, such as *Achromobacter* species, lineages of the *Burkholderia cepacia* complex and *Burkholderia gladioli*, have rarely been described as the cause of PD-related peritonitis^32^. The precise identification of NF-GNB is a challenge for conventional microbiology due to the phenotypic similarity and taxonomic complexity of these agents. Phenotypic tests based on morphology and biochemical characteristics often provide erroneous identification of these species^33^. In our study, such limitations were minimized with the identification of the isolates by MALDI-TOF, a method based on the identification of bacterial ribosomal proteins (very abundant and essential for microorganism survival), whose accuracy of identification is improved when compared to phenotypic tests, which can sometimes present atypical or delayed reactions. For NF-GNB isolates, MALDI-TOF has a great advantage in identifying these organisms, which are hard to correctly identify by conventional methods^34^. This identification technique was mentioned in the most recent ISPD guideline on PD-related peritonitis, although at the time of its publication, there was insufficient evidence for its recommendation^23^.

Our study showed that preexisting ESI was the only independent predictor of nonresolution. The association between ESI and subsequent peritonitis is widely recognized^35^. *Staphylococcus aureus* and *Pseudomonas aeruginosa* are the most common microorganisms causing ESI, and they can tunnel along the subcutaneous pathway and lead to peritonitis^35^. On the other hand, only a few publications have focused on the influence of ESI on peritonitis treatment response^36^, reporting that the presence of this infection is associated with poor outcomes. According to Gupta et al.^37^, in such cases, antibiotics do not resolve peritoneal infection, although transient clearing of the effluent may occur.

Of note, in this study, biofilm production did not influence the outcome; however, this result does not rule out the possibility that biofilms, in concert with other virulence factors, may influence peritonitis evolution.

Our study has several limitations, the most important being the small sample size, aggravated use of different antibiotic regimens, and the impossibility of recovering approximately 20% of the isolates. In addition, we do not currently have data on the production of virulence factors by bacteria other than biofilms. Beyond that, we did not perform tunnel sonography in patients, which limits the diagnosis of tunnel infection. Finally, there is a lack of information on patients’ nutritional status, comorbidities other than diabetes and comorbidity scores. However, this is a study involving different NF-GNB species and therefore allows comparisons between peritonitis episodes due to *Pseudomonas* species and those due to other species. To our knowledge, this is the first study to address the role of biofilm bacterial production on the outcomes of NF-GNB-induced peritonitis in PD patients. However, it must be considered that the formation of biofilms is a complex process that involves not only the production of biofilm components by bacteria but also host cell/tissue interactions, PD time, and other underrecognized factors^38^.

This study reported a considerably high prevalence of multiresistant *Acinetobacter* species causing PD-related peritonitis, which raises concern about care for the prevention and management of these infections. In addition, it provided novel information about pathogens that cause peritonitis, including species of *Achromobacter*, suggesting that there is some benefit of new techniques such as MALDI-TOF in identifying the causative organism in PD-related peritonitis.

Finally, the prevalence of PD-related NF-GNB in our center was similar to that observed in BRAZPD, the largest Latin American cohort of incident PD patients, and again highlighted the severity of these infections.

## Methods

This retrospective study was conducted in accordance with the Declaration of Helsinki and approved by the Botucatu Medical School’s Research Ethics Committee.

We reviewed all episodes of PD-related peritonitis caused by NF-GNB that occurred between June 1997 and December 2015 in a single Brazilian university center. The exclusion criteria were episodes with incomplete clinical data, relapse (episodes caused by the same species or a negative culture result within 28 days of completion of antibiotic therapy), recurrence (episodes caused by other species within 28 days after starting antibiotic therapy), and repeat episodes (episodes caused by the same species or after 28 days following completion of antibiotic therapy).

The diagnosis of peritonitis was made when at least two of the following criteria were present: the presence of a cloudy peritoneal effluent; abdominal pain; dialysate containing more than 100 leukocytes per microliter (at least 50% polymorphonuclear cells); and positive culture of dialysate^23^. The outcomes were defined as follows: resolution (disappearance of signs and symptoms within 5 days after the initiation of antibiotic therapy); relapse, refractory peritonitis (presence of turbid dialysate after 5 days of treatment with appropriate antibiotics); peritonitis-related death (death of a patient with active peritonitis or the death of a patient who had an episode within the previous 4 weeks)^23^; and nonresolution (catheter removal before the 5th day of treatment, refractory peritonitis, relapse, or peritonitis-related death).

### Catheter insertion and care and dialysis procedures

The catheter placements were made under supervision of a senior nephrologist with percutaneous blind insertion of a double cuff straight Tenckhoff catheter using the Seldinger technique. Until 2003, no patients used antibiotic cream application at the catheter exit site; from 2003 to 2006, we prescribed daily mupirocin cream, and from January 2007, daily gentamicin was prescribed to all incident patients. All patients used a semiocclusive dressing with sterile gauze and microporous adhesive tape.

Until 1999, the CAPD connection systems were the Y set type; the twin bag was introduced in 1999. APD was introduced in 1998, and its indication and prescription were based on clinical criteria or the patient’s preference. For both PD modalities, we used standard glucose solutions with low pH and high glucose degradation product levels. No patients were treated with icodextrin dialysis solution.

The diagnosis of ESI followed the International Society for Peritoneal Dialysis (ISPD) criteria, such as the presence of purulent discharge, with or without erythema of the skin at the catheter–epidermal interface^39^.

### Data collection

We recorded the following information for each case: date, preexisting ESI (ESI diagnosed within four weeks before a peritonitis episode), tunnel infection, topical antibiotic use at the catheter exit site, initial antimicrobial treatment for peritonitis and subsequent adjustments, outcome, treatment time before the peritonitis episode (dialysis vintage), patient characteristics (age, sex, race [Caucasian or non-Caucasian]), underlying kidney disease, previous peritonitis by other bacteria, PD modality (continuous ambulatory PD or automated PD), and characteristics of the causative germ (species, biofilm production capability, and in vitro antibiotic susceptibility).

### Microbiological routine diagnosis

After peritonitis diagnosis, each dialysate sample was processed following the recommendations of the ISPD^23^. Cultures were performed using rapid blood culture bottle kits of the Bactec System (BECTON AND DICKINSON COMPANY-BD, Brazil). The proportion of strains susceptible to each drug was defined based on the 2016 Clinical Laboratory Standards Institute breakpoints^40^. After isolation, identification, and routine susceptibility testing using broth antibiogram, strains were stored in a culture collection at − 70 °C.

### Special microbiological tests

The stored samples were reisolated on MacConkey agar plates and reidentified. For this, isolates were gram-stained to confirm purity and to determine each isolate’s morphology and specific color. Afterward, the isolates were identified by conventional biochemical testing^39,41^ and by mass spectrometry using MALDI-TOF (matrix-assisted laser desorption ionization time-of-flight) technology (VITEK, Brazil)^42^.

### In vitro susceptibility

The in vitro susceptibility to amikacin, ciprofloxacin, cefepime, imipenem, and ceftazidime was determined by the minimum inhibitory concentration (MIC) based on gradient diffusion using the E test (BIOMERIEUX, INC. USA). The proportion of strains susceptible to each drug was defined based on the 2016 Clinical Laboratory Standards Institute breakpoints^40^. When strains presented intermediate MIC values, we considered them resistant.

### Biofilm production

The bacterial samples were grown in tryptic soy broth (TSB) (BECTON AND DICKINSON COMPANY-BD, Brazil) at 37 °C for 18 h. To assess the bacterial ability to adhere to abiotic surfaces, we used 96-well polystyrene plates and added 200 µL of TSB and 10 µL of the bacterial suspension (approximately 108 CFU/mL) to each well, except one well that was inoculated only with culture medium to be used as a reading standard (blank). The plates were incubated at 37 °C for 48 h and then washed with phosphate-buffered saline 4 times to remove nonadherent bacteria. Bacteria that adhered to the abiotic surface were then fixed with formalin (2%), and after 20 min, the formalin was removed, and the preparations were washed 4 more times with water. Then, the preparations were stained with a crystal violet solution (1%) for 20 min, after which they were washed 3 times with water to remove excess dye. After drying, the dye was solubilized with methanol for 10 min, and the optical density, measured at 540 nm, was determined^43^. Then, we classified biofilm production into one of four categories as previously published^43^: no producer, weak producer, moderate producer, and strong producer. In our study, we opted for a 48-h method instead of faster methods to obtain a more reliable result, as it allows the strains to have enough time for the production of biofilms because some NF-GNB species show slow growth.

### Clinical-microbiological associations

Each patient’s characteristics, preexisting ESI, topical antibiotic use at the catheter exit site, initial treatment for peritonitis, treatment adjustment after bacterial identification and in vitro susceptibility, previous peritonitis by other bacteria, and microbiological properties were analyzed regarding their association with the outcome.

### Statistical analysis

For comparison between frequencies, we used the chi-squared test or Fisher’s exact test. Binary logistic regression with a backward stepwise procedure was used to determine the independent predictors of outcomes. For this purpose, we first performed a univariate logistic regression analysis to select the variables that would enter the final model, with *p* > 0.20 as the elimination criterion. Collinearity among variables was tested, and if statistically significant interactions occurred, one of the variables was excluded. A *p* value < 0.05 was considered significant.

### Ethics approval

At the beginning of regular PD treatment, an informed consent was obtained from all participants and/or their legal guardian/s for the use of their clinical and laboratory data for research purposes. Therefore, the Botucatu Medical School’s Research Ethics Committee approved this study (CAAE 64736017.2.0000.5411 statement) and exempted it of any specific informed consent.

## References

[1] Moncrief, J.W., Popovich, R.P. Continuous ambulatory peritoneal dialysis (CAPD)—worldwide experience in peritoneal dialysis. In *Developments in Nephrology*, vol 2. (ed. Nolph K.D.) 178–212 (Springer, 1981).

[2] Popovich RP, Moncrief JW, Nolph KD, Ghods AJ, Twardowski ZJ, Pyle WK (1978). Continuous ambulatory peritoneal dialysis. Ann. Intern. Med..

[3] Fenton SS, Cattran DC, Allen AF, Rutledge P, Ampil M, Dadson J, Locking H, Smith SD, Wilson DR (1979). Initial experiences with continuous ambulatory peritoneal dialysis. Artif. Organs..

[4] Oreopoulos DG, Robson M, Izatt S, Clayton S, deVeber GA (2009). A simple and safe technique for continuous ambulatory peritoneal dialysis (CAPD). BMC Infect Dis.

[5] Buoncristiani U (1996). Birth and evolution of the "Y" set. ASAIO J..

[6] Piraino B, Bernardini J, Florio T, Fried L (2003). Staphylococcus aureus prophylaxis and trends in Gram negative infections in peritoneal dialysis patients. Perit. Dial. Int..

[7] de Moraes TP, Figueiredo AE, de Campos LG, Olandoski M, Barretti P, Pecoits-Filho R, BRAZPD Investigators (2014). Characterization of the BRAZPD II cohort and description of trends in peritoneal dialysis outcome across time periods. Perit. Dial. Int..

[8] Pecoits-Filho R, Yabumoto FM, Campos LG, Moraes TP, Figueiredo AE, Olandoski M, Shimakura SE, Barretti P, BRAZPD Investigators (2018). Peritonitis as a risk factor for long-term cardiovascular mortality in peritoneal dialysis patients: the case of a friendly fire?. Nephrology.

[9] Bunke CM, Briel MR, Golper TA (1997). Outcomes of single organism peritonitis in peritoneal dialysis: gram negatives versus gram positives in the Network 9 Peritonitis Study. Kidney. Int..

[10] Troidle L, Gorban-Brennan N, Kliger A, Finkelstein F (1998). Differing outcomes of gram-positive and gram-negative peritonitis. Am. J. Kidney. Dis..

[11] Siva B, Hawley CM, McDonald SP, Brown FG, Rosman JB, Wiggins KJ, Bannister KM, Johnson DW (2009). Pseudomonas peritonitis in Australia: predictors, treatment, and outcomes in 191 cases. Clin. J. Am. Soc. Nephrol..

[12] Lu W, Kwan BC, Chow KM, Pang WF, Leung CB, Li PK, Szeto CC (2018). Peritoneal dialysis-related peritonitis caused by *Pseudomonas* species: Insight from a post-millennial case series. PLoS ONE.

[13] Li PH, Cheng VCC, Yip T, Yap DYH, Lui S-L, Lo WK (2017). Epidemiology and clinical characteristics of Acinetobacter peritoneal dialysis-related peritonitis in Hong Kong—with a perspective on multi-drug and carbapenem resistance. Perit. Dial. Int..

[14] de Moraes TP, Olandoski M, Caramori JC, Martin LC, Fernandes N, Divino-Filho JC, Pecoits-Filho R, Barretti P (2014). Novel predictors of peritonitis-related outcomes in the BRAZPD cohort. Perit. Dial. Int..

[15] Barretti P, Pereira D, Brasil MA, de Lourdes Cunha M, Caramori J, Montelli A (2009). Evolution of gram-negative bacilli susceptibility in peritoneal dialysis-related peritonitis in Brazil: a single center’s experience over nine years. Perit. Dial. Int..

[16] Cepas V, Soto SM (2020). Relationship between Virulence and Resistance among Gram-Negative Bacteria. Antibiotics..

[17] Harper D, Parracho HM, Walker J, Sharp R, Hughes G, Werthén M, Lehman S, Morales S (2014). Bacteriophages and biofilms. Antibiotics.

[18] Simpson JA, Smith SE, Dean RT (1988). Alginate inhibition of the uptake of Pseudomonas aeruginosa by macrophages. J. Gen. Microbiol..

[19] Wei Q, Ma LZ (2013). Biofilm matrix and its regulation in Pseudomonas aeruginosa. Int. J. Mol. Sci..

[20] Doughari HJ, Ndakidemi PA, Human IS, Benade S (2011). The ecology, biology and pathogenesis of *Acinetobacter* spp.: an overview. Microbes Environ..

[21] Martins VV, Pitondo-Silva A, de Manço LM, Falcão JP, dos Freitas SS, da Silveira WD, Stehling EG (2014). Pathogenic potential and genetic diversity of environmental and clinical isolates of *Pseudomonas aeruginosa*. APMIS.

[22] Visca P, Seifert H, Towner KJ (2011). Acinetobacter infection—an emerging threat to human health. IUBMB Life.

[23] Li PK, Szeto CC, Piraino B, de Arteaga J, Fan S, Figueiredo AE, Fish DN, Goffin E, Kim YL, Salzer W, Struijk DG, Teitelbaum I, Johnson DW (2016). ISPD Peritonitis Recommendations: 2016 update on prevention and treatment. Perit. Dial. Int..

[24] Barretti, P., Montelli, A. C., Batalha, J. E., Caramori, J. C. & Cunha, M. L. The role of virulence factors in the outcome of staphylococcal peritonitis in CAPD patients. *BMC Infect Dis.* (2009). 10.1186/1471-2334-9-212 PMC2807432 20028509

[25] Barretti P, Moraes TM, Camargo CH, Caramori JC, Mondelli AL, Montelli AC, da Cunha ML (2012). Peritoneal dialysis-related peritonitis due to *Staphylococcus aureus*: a single-center experience over 15 years. PLoS ONE.

[26] Dias RCB, Vieira MA, Moro AC, Ribolli DFM, Monteiro ACM, Camargo CH, Tiba-Casas MR, Soares FB, Dos Santos LF, Montelli AC, da Cunha MLRS, Barretti P, Hernandes RT (2019). Characterization of *Escherichia coli* obtained from patients undergoing peritoneal dialysis and diagnosed with peritonitis in a Brazilian centre. J. Med. Microbiol..

[27] Tetz GV, Artemenko NK, Tetz VV (2009). Effect of DNase and antibiotics on biofilm characteristics. Antimicrob. Agents Chemother..

[28] Badamchi A, Masoumi H, Javadinia S, Asgarian R, Tabatabaee A (2017). Molecular detection of six virulence genes in *Pseudomonas aeruginosa* isolates detected in children with urinary tract infection. Microb. Pathog..

[29] Costerton W, Lewandowski Z, Caldwell DE, Korber DR, Lappin-Scott HM (1995). Microbial biofilms. Annu. Rev. Microbiol..

[30] Chao CT, Lee SY, Yang WS, Chen HW, Fang CC, Yen CJ, Chiang CK, Hung KY, Huang JW (2014). Acinetobacter peritoneal dialysis peritonitis: a changing landscape over time. PLoS ONE.

[31] Aliramezani A, Soleimani M, Fard RMN, Nojoomi F (2019). Virulence determinants and biofilm formation of *Acinetobacter baumannii* isolated from hospitalized patients. Germs..

[32] Apostolovic BL, Velickovic-Radovanovic RM, Andjelkovic-Apostolovic MR, Cvetkovic TP, Dinic MM, Radivojevic JD (2015). Repeated Burkholderia cepacia peritonitis in a patient undergoing continuous ambulatory peritoneal dialysis. West Indian Med. J..

[33] Cankaya E, Keles M, Gulcan E, Uyanik A, Uyanik H (2014). A rare cause of peritoneal dialysis-related peritonitis: Achromobacter denitrificans. Perit. Dial. Int..

[34] Cloud JL, Harmsen D, Iwen PC, Dunn JJ, Hall G, Lasala PR, Hoggan K, Wilson D, Woods GL, Mellmann A (2010). Comparison of traditional phenotypic identification methods with partial 5' 16S rRNA gene sequencing for species-level identification of nonfermenting Gram-negative bacilli. J. Clin. Microbiol..

[35] Piraino B, Bernardini J (2013). Catheter-related peritonitis. Perit. Dial. Int..

[36] Gupta B, Bernardini J, Piraino B (1996). Peritonitis associated with exit site and tunnel infections. Am. J. Kidney Dis..

[37] Bunke CM, Brier ME, Golper TA (1997). Outcomes of single organism peritonitis in peritoneal dialysis: gram negatives versus gram positives in the Network 9 Peritonitis Study. Kidney Int..

[38] Martins M, Rodrigues A, Pedrosa JM, Carvalho MJ, Cabrita A, Oliveira R (2013). Update on the challenging role of biofilms in peritoneal dialysis. Biofouling.

[39] CLSI. Performance Standards for Antimicronial Susceptibility Testing. 29th ed. CLSI supplement M100S. https://clsi.org/media/2663/m100ed29_sample.pdf (accessed 17 September 2020).

[40] Szeto CC, Li PK, Johnson DW, Bernardini J, Dong J, Figueiredo AE, Ito Y, Kazancioglu R, Moraes T, Van Esch S, Brown EA (2017). ISPD Catheter-Related Infection Recommendations: 2017 Update. Perit. Dial. Int..

[41] Koneman, E. W. & Winn, W. C. Koneman’s Color Atlas and Textbook of Diagnostic Microbiology, 6th (eds. Winn, S. *et al.*) 380–382 (Lippincott Williams & Wilkins, 2006)

[42] Dubois D, Grare M, Prere MF, Segonds C, Marty N, Oswald E (2012). Performances of the Vitek MS matrix-assisted laser desorption ionization-time of flight mass spectrometry system for rapid identification of bacteria in routine clinical microbiology. J. Clin. Microbiol..

[43] Stepanovic S, Vukovic D, Dakic I, Savic B, Svabic-Vlahovic M (2000). A modified microtiter-plate test for quantification of staphylococcal biofilm formation. J. Microbiol. Methods..

